# Amniotic fluid from healthy term pregnancies does not harbor a detectable microbial community

**DOI:** 10.1186/s40168-018-0475-7

**Published:** 2018-05-11

**Authors:** Efrem S. Lim, Cynthia Rodriguez, Lori R. Holtz

**Affiliations:** 1 School of Life Sciences, Arizona State University, Tempe, AZ 85287 USA; 2 Center for Fundamental and Applied Microbiomics, The Biodesign Institute, Tempe, AZ 85287 USA; 3 Department of Pediatrics, Washington University School of Medicine, 660 S. Euclid Ave., Campus Box 8208, St. Louis, MO 63110 USA

**Keywords:** Amniotic fluid, Microbiome, Virome, Sterile body fluid, Virus, Bacteria, Microbial invasion of the amniotic cavity

## Abstract

Recent studies have conflicting data regarding the presence of intra-amniotic microbiota. Viral communities are increasingly recognized as important although overlooked components of the human microbiota. It is unknown if the developing fetus is exposed to a community of viruses (virome). Given the debate over the existence of an intra-amniotic microbial community and the importance of understanding how the infant gut is populated, we characterized the virome and bacterial microbiota of amniotic fluid from 24 uncomplicated term pregnancies using next-generation sequencing methods. Contrary to expectations, the bacterial microbiota of amniotic fluid was indistinguishable from contamination controls. Viral reads were sparse in the amniotic fluid, and we found no evidence of a core viral community across samples.

**Electronic supplementary material:**

The online version of this article (10.1186/s40168-018-0475-7) contains supplementary material, which is available to authorized users.

## Introduction

Microbial communities play an important role early in the life of infant development by influencing nutritional and immune functions [1]. Because factors that influence the composition of the early gut microbiota have significant prognostic and therapeutic implications, there is intense interest in understanding if microbial interactions occur within the fetal environment and, if so, how they impact maternal and fetal health.

Amniotic fluid has traditionally been viewed as a sterile site [2]. In healthy pregnancies, culture-based studies of mid-trimester amniotic fluid obtained for genetic testing have either found amniotic fluid to be sterile [3–5] or only isolated bacteria in up to 13% of amniotic fluid samples [2, 6–8]. Similarly, targeted and broad range molecular methods to detect bacterial agents in mid-trimester amniotic fluid also either did not detect bacteria [9] or detected bacteria in only up to 11% of samples [10, 11]. However, bacteria can be detected in amniotic fluid from pregnancies with complications such as preterm labor [12], preeclampsia [13], small for gestational age [14], and preterm prelabor rupture of membranes [15] by culture- and molecular-based methods. Recently, using next-generation sequencing, bacterial sequences were detected in amniotic fluid from 15 healthy term gestations [16], suggesting that human amniotic fluid harbors a microbial community. The detection of microbial populations in healthy term amniotic fluid implies that neonatal microbial colonization, and thus microbial influences on infant health and development, begins prior to birth.

Likewise, there is contention about the existence of bacterial populations in other components of the in utero environment including the placenta, cord blood, and meconium [17, 18]. Bacteria have been isolated by culture [19] and visualized [20] in 21–26% of placentas from healthy term deliveries. Viable bacteria (*Enterococcus*, *Streptococcus*, *Staphylococcus*, or *Propionibacterium*) have also been isolated from the cord blood of healthy newborns [21]. With the advent of next-generation sequencing, bacterial sequences have also been detected in the placenta [16, 22] and meconium [16, 23, 24] from term gestations. However, a recent study found that the bacterial microbiota of term placentas resembled that of contamination and extraction controls [25]. Furthermore, since gnotobiotic animals from many mammalian species can reliably be derived via sterile cesarean section, it is reasoned the in utero environment of healthy pregnancies is sterile [17].

Considerably less is known about viruses in the fetal environment. Specific pathogenic viruses such as cytomegalovirus (CMV), HIV, enteroviruses, influenza, rubella, varicella, Zika, and human papilloma viruses (HPV) are certainly transmitted transplacentally or vaginally to the fetus. Using targeted PCR, CMV, adenovirus, herpes simplex virus, human herpesvirus 6, enterovirus, Epstein-Barr virus, respiratory syncytial virus, and human parvovirus B19 can be detected in amniotic fluid obtained via amniocentesis for genetic screening [26–30], apparently co-existing with the fetus without causing symptoms. Viruses are infrequently detected in amniotic fluid from a variety of disease states using PCR for a panel of eukaryotic viruses followed by mass spectrometry (PCR/ESI-MS) including 1.4% of amniotic fluid from women with preterm labor and intact membranes [31], 1.7% of amniotic fluid from cases of preterm prelabor rupture of membranes [32], and 3% of samples from women with sonographic short cervix [33]. Additionally, early in life stools (first 96 h of life) from term infants have both eukaryotic viruses and bacteriophage present [34]. However, there are no studies using an unbiased approach to determine if viruses are present in amniotic fluid.

Here, we ask using sequence-based methods if term gestations have an amniotic fluid bacterial community and/or virome (community of eukaryotic viruses and bacteriophages), which would have major implications on our understanding of how the infant gut is initially populated by microbes.

## Results

### Amniotic fluid has low bacterial biomass

We evaluated 24 amniotic fluid specimens from term uncomplicated pregnancies obtained at the time of elective cesarean section at Barnes-Jewish Hospital, St. Louis, Missouri. The median maternal age was 28 years, and the median infant weight at birth was 3288 g (Additional file 1: Table S1 and Additional file 2).

**Fig. 1:**
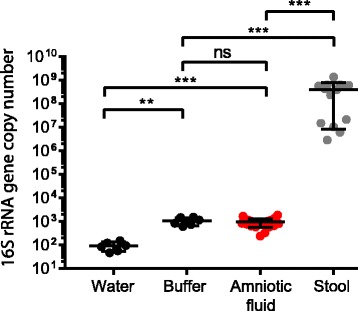
Bacterial 16S rRNA gene quantitative PCR. 16S rRNA gene copies per reaction were quantified in the amniotic fluid samples, water (reagent negative control), buffer (extraction negative control), and pediatric stool samples. Statistical significance was assessed by Mann-Whitney test; **P ≤ 0.01, ***P ≤ 0.0001. ns, non-significant

To quantify the bacterial biomass in amniotic fluid specimens, we performed a qPCR assay to measure the absolute number of 16S rRNA gene copies. The mean 16S rRNA gene copies in amniotic fluid samples were 944 copies/mL (SD 374), a very low bacterial biomass (Fig. 1) when compared to pediatric stool samples which was 3.92 × 10^8^ copies/g (SD 3.84 × 10^8^). We were concerned that these low-density sequences reflected contamination. Therefore, we sought the 16S rRNA gene copies in negative controls that were performed in parallel: DNA-free water (reagent negative control) that had no additional input material and did not undergo the DNA extraction protocol and extraction buffer that was subjected to the same DNA extraction protocol used for the samples (extraction negative control). The mean 16S rRNA gene copies in water (negative control) were 93 copies (SD 40), and the mean 16S rRNA gene copies in buffer extraction negative controls were 1062 copies/mL (SD 390). While amniotic fluid 16S rRNA gene copies were higher than the water negative controls, there was no statistically significant difference between the 16S rRNA gene copy number between amniotic fluid and buffer extraction negative controls. Thus, consistent with prior studies [9–11], amniotic fluid had an absolute 16S rRNA gene copy number indistinguishable from the extraction negative controls.

### The bacterial microbiota signature of amniotic fluid is indistinguishable from controls

**Fig. 2:**
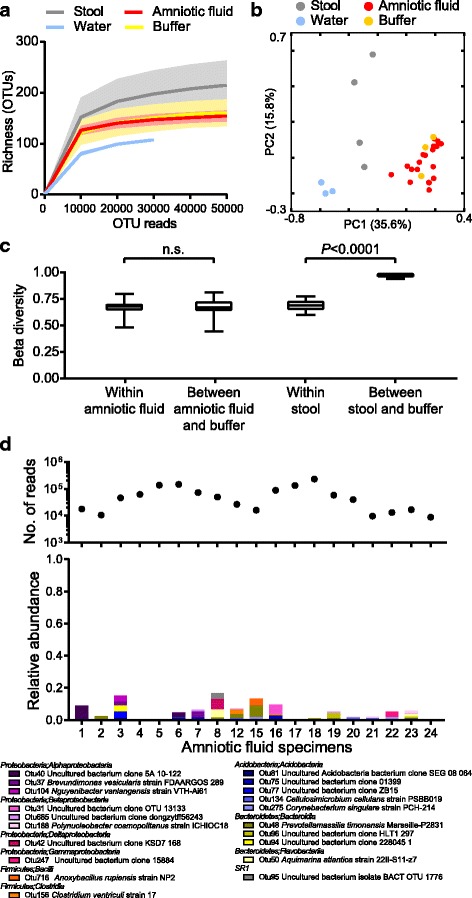
The bacterial microbiota of amniotic fluid is indistinguishable from controls. **a** Richness (number of bacterial OTUs) of each sample type by sequencing depth. **b** PCoA of unweighted UniFrac distances. **c** Bray-Curtis dissimilarity analysis compared within sample type and between sample type and buffer control. Statistical significance was assessed by Mann-Whitney test. **d** Relative abundance of bacterial OTUs unique to amniotic fluid and not present in negative controls

We next considered the possibility that amniotic fluid harbored a qualitatively different set of bacterial DNA than the reagents, i.e., that there was discrete bacterial microbiota present at low abundance in amniotic fluid. If so, bacteria of true amniotic fluid origin should differ in content from the negative controls. Therefore, we performed deep sequencing of the 16S rRNA gene V4 region from amniotic fluid, water (reagent negative control), buffer (extraction negative control), and pediatric stool samples (positive control) Additional file 3. Seven samples were omitted from further analyses as they contained less than 5000 16S rRNA gene sequencing reads (five amniotic fluids, one water, and one buffer). We found that there was no statistically significant difference between the bacterial richness of amniotic fluid specimens and buffer extraction negative control (Fig. 2a). Amniotic fluid bacterial richness was higher than water negative controls, but lower than the stool controls. To compare the bacterial community structure between the amniotic fluid specimens and controls, we measured the unweighted UniFrac distances between the samples and performed PCoA analyses. Amniotic fluid specimens overlapped extensively with buffer extraction negative controls but were distinct from stool (positive controls) and water negative controls (Fig. 2b). This was confirmed by Bray-Curtis dissimilarity analyses: although the bacterial microbiota of stool had significantly higher dissimilarities from buffer controls than when compared to other stools (Fig. 2c, right), we found no statistically significant difference between the bacterial microbiota detected in amniotic fluid and buffer controls (Fig. 2c, left).

We were concerned that a limitation of diversity measurements (Fig. 2b, c) is their lack of sensitivity for rare taxa, such as rare OTUs within a dominant background of contamination-derived OTUs. Therefore, we sought to identify bacterial OTUs that were present in amniotic fluid but absent from buffer and water negative controls Additional file 4. We found that bacterial OTUs unique to amniotic fluid accounted for very little of the relative abundance (Fig. 2d). Importantly, these rare bacterial OTUs were not frequently detected across the other amniotic fluid specimens.

### Viruses are rarely detected in amniotic fluid

The emerging “intra-amniotic microbiome” hypothesis raises the possibility that amniotic fluid might also harbor a resident community of viruses. Thus, we investigated the virome of these amniotic fluid specimens. To comprehensively detect both DNA and RNA viruses, total nucleic acid extracted from amniotic fluid specimens was subjected to sequence-independent DNA and RNA amplification (SIA), which provides an unbiased representation of RNA viruses and, to a lesser degree, DNA viruses. Likewise, water reagent negative controls and buffer extraction negative controls (PBS subjected to the same sample processing and nucleic acid extraction protocols as samples) were included in these experiments. Only one amniotic fluid specimen yielded a eukaryotic virus—GB virus C (specimen #10, 12 viral sequencing reads). GB virus C, also known as human pegivirus, is an RNA virus that is detected in approximately 2–4% of blood donors [35].

**Fig. 3:**
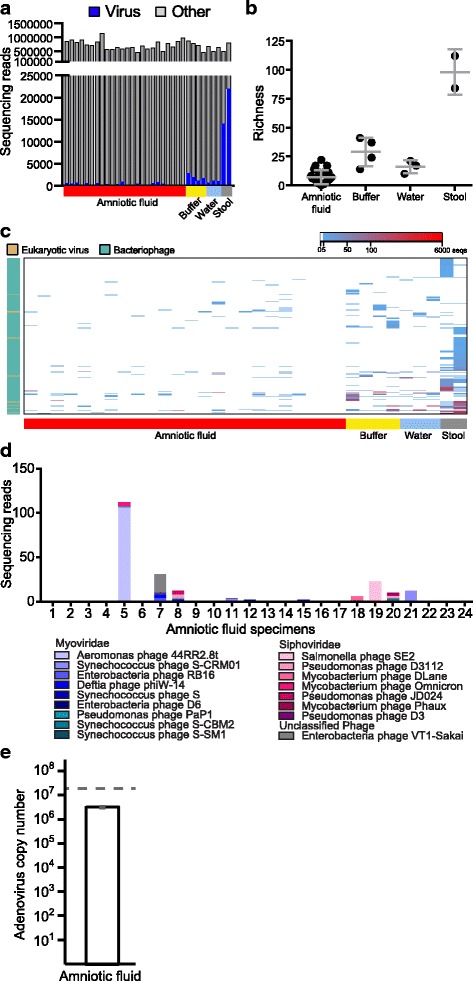
Viruses are rarely detected in amniotic fluid. **a** Sequencing reads (virus and other) by sample type. **b** Viral richness by sample type. **c** Heatmap of reads assigned to virus species. **d** Number of reads assigned to viral species per sample which were unique to amniotic fluid and not present in negative controls. **e** Amount of adenovirus plasmid detected in spiked amniotic fluid sample and amount in spiked material (dotted line)

Because we only identified a single RNA virus, we then used multiple displacement amplification (MDA) which only identifies DNA viruses and is generally more sensitive for their detection than the SIA method [34]. The average number of viral sequencing reads in amniotic fluid was 331 reads (SD 248) (Fig. 3a). In comparison, stool specimens yielded an average of 34,027 viral sequencing reads (SD 44,667). The viral richness of amniotic fluid specimens was less than buffer extraction negative controls, water reagent negative controls, and stool samples (Fig. 3b) Additional file 5. Most of the viruses detected in amniotic fluid could be attributed to the extraction negative controls and/or water reagent negative controls (Fig. 3c). We next sought to identify viruses that were detected only in amniotic fluid specimens, but absent from controls. We found that one amniotic fluid specimen (specimen 5) had 106 sequencing reads of a bacteriophage most similar to the Aeromonas phage 44RR2.8t. However, most other amniotic fluid specimens did not have “amniotic fluid unique” viral sequences (Fig. 3d). To evaluate whether inhibitors of PCR might be present in these amniotic fluid specimens, we performed a spike-in experiment. 1.8 × 10^7^ copies of a plasmid containing a portion of the adenovirus hexon gene were added to an amniotic fluid specimen, followed by total nucleic acid extraction. Quantitative PCR targeting the spiked nucleic acid detected 3.3 × 10^6^ copies (Fig. 3e), indicating that the paucity of viruses in amniotic fluid was not due to PCR inhibition.

## Discussion

These data fail to identify a population of bacterial microbiota in amniotic fluid from healthy term pregnancies that meaningfully differs in concentration or content from the sequences amplified from negative controls (Figs. 1 and 2). The most parsimonious explanation for our inability to find differences is that amniotic fluid of healthy term pregnancies has negligible bacterial biomass. Similarly, we find only limited evidence for viral presence using metagenomic sequencing of material that has been subjected to preparation techniques optimized to recover DNA as well as RNA viruses, including DNA and RNA bacteriophages. Based on these analyses, we provisionally conclude that the term infant is not normally exposed to bacterial or viral populations in the immediate pre-birth interval.

Although the womb is traditionally viewed as sterile, recent molecular findings of bacterial DNA in the in utero environment challenge this paradigm [16, 22, 36]. Since the intra-amniotic cavity is distinct from the placenta, we cannot discount the possibility that the placenta is separately colonized with microbes and that perhaps protective mechanisms prevent entry of these agents into the amniotic fluid. We also acknowledge that our data pertain only to amniotic fluid at term and that there might have been earlier in gestation colonization with bacteria and/or viruses. Indeed, alteration of the placental and amniotic fluid bacterial microbiota has been associated with preterm birth [12, 20, 37, 38]. Our work further demonstrates that when sequencing samples with low microbial density, it is critical to pay assiduous attention to controls, so as not to attribute microbial presence in specimens to contamination [25]. Our findings also prompt intensive efforts to learn how the chorioamniotic unit is so efficient at preventing colonization of the amniotic fluid despite evidence for circulating bacteria and viruses in healthy adults [39–41].

## Methods

### Subjects

This study was approved by the Human Research Protection Office of Washington University School of Medicine in St. Louis. Twenty-four archived frozen amniotic fluid samples were obtained from the Women and Infants Health Specimen Consortium biobank at Washington University. Samples were selected if non-laboring, C-section, and full-term gestation. Women with diabetes of any type, hypertension of any type, seizure disorder, intrauterine growth restriction, cancer, heart disease, kidney disease, or treated with acyclovir were excluded.

### Sample collection

Amniotic fluids were obtained in a sterile fashion at the time of C-section by aspirating through intact amniotic membranes. The amniotic fluid was then spun at 1620*g* for 5 min at 4 °C. Fluid was then placed into conical tubes and stored at − 80 °C.

### Bacterial 16S rRNA gene sequencing

One thousand fifty microliters of amniotic fluid was centrifuged (7000*g*, 10 min). Extraction buffer was added to the pellet, which was disrupted by bead beating, and DNA was extracted using QIAamp DNA stool Mini Kit in a decontaminated sterile environment. In parallel, four buffer-only blank controls and four pediatric stool samples were disrupted by bead beating and extracted to serve as negative and positive controls respectively. PCR was performed using Golay-barcoded primers specific for the V4 region (F515/R806). Reactions were held at 94 °C for 2 min to denature the DNA, with amplification proceeding for 40 cycles at 94 °C for 15 s, 50 °C for 30 s, and 68 °C for 30 s, and a final extension of 2 min at 68 °C. Each sample was amplified in triplicate, combined, and cleaned using Ampure bead clean up kit. Equimolar libraries were pooled and sequenced using an Illumina MiSeq sequencer (2 × 250 v2 kit) at the Center for Genome Sciences & Systems Biology at Washington University.

### Bacterial 16S rRNA gene analysis

16S OTU clustering was performed using UPARSE (http://drive5.com/uparse/) [42]. Paired reads were merged and filtered at maximum expected error threshold of 1.0 (-fastq_maxee 1.0). Unique sequences were identified using the “fastx_uniques” command, followed by clustering at 97% and chimera filtering using “cluster_otus”. For OTU identification, the 16S rRNA gene sequences were subjected to BLASTN search against the NCBI 16S ribosomal RNA database. Ecological analyses were performed using the vegan package in R and QIIME2 scripts.

### Bacterial 16S rRNA gene qPCR

SYBR green quantitative PCR for 16S rRNA gene was performed using primers 515F (5′-GTGCCAGCMGCCGCGGTAA-3′) and 805R (5′-GACTACCAGGGTATCTAATCC-3′) primers on DNA as previously described [43]. The qPCR was performed using TaqMan Fast Advanced Master Mix (Thermo Fisher). The 25-μL reaction included 5 μL of extracted DNA and 5 μmol of each primer. The following cycling conditions were used: 95 °C for 10 min, then 40 cycles of 95 °C for 15 s and 60 °C for 60 s followed by a melt curve. To generate a standard curve for this assay, a plasmid containing the 16S PCR amplicon from *Escherichia coli* (DH5α) was serially diluted from 5 × 10^7^ copies to five copies and used to generate a standard curve; a limit of detection of 500 copies was defined. Samples were tested in a 96-well plate format with six water-only negative controls, six buffer only controls, and 14 pediatric stools samples. All water controls were below the limit of detection.

### Virome sequencing

One thousand fifty microliters of amniotic fluid was centrifuged (7000*g*, 10 min). The supernatant was then filtered through a 0.45-μm membrane. Total nucleic acid was extracted from the filtrate using COBAS Ampliprep (Roche). In parallel, PBS was filtered and extracted to serve as extraction reagent-only control and two pediatric stool samples were filtered and extracted to serve as a positive control. Sequence-independent DNA and RNA amplification (SIA) was performed on the total nucleic acid as previously described [34] and used for NEBNext library construction (Illumina). For multiple displacement amplification (MDA), total nucleic acid and three water negative controls were amplified with Phi29 polymerase (GenomiPhi V2 kit, GE Healthcare) according to the manufacturer’s instructions and used for Nextera DNA library construction (Illumina). Libraries were purified and size-selected using Agencourt Ampure XP beads (Beckman-Coulter), followed by quantification using a 2100 Bioanalyzer (Agilent Technologies). Multiplexed SIA libraries were pooled and sequenced separately from multiplexed MDA libraries.

### Virome sequence analysis

Illumina sequencing reads were analyzed using VirusSeeker [44], a BLAST-based computational pipeline to identify viral sequences. Taxonomic classification for bacteriophage sequences were parsed using MEGAN version 6.10.2 [45]. Ecological analyses were performed using the vegan package in R and QIIME2 scripts. Sequencing reads were normalized to 400,000 reads per sample.

### Spiking of amniotic fluid

To determine whether inhibitors of PCR might be present in these amniotic fluid specimens, 1.8 × 10^7^ copies of a plasmid containing a portion of the adenovirus hexon gene were added to 1050 μL of amniotic fluid and to 1050 μL of PBS. Total nucleic acid was extracted from these spiked samples using COBAS Ampliprep (Roche). A previously published qPCR targeting the adenovirus hexon gene was then used to quantify the spiked DNA [46]. The qPCR was performed using TaqMan Fast Advanced Master Mix (Thermo Fisher). The 20-μL reaction included 5 μL of extracted total nucleic acid, 18 pmol of each primer, and 5 pmol of probe. The following cycling conditions were used: 95 °C for 2 min, then 45 cycles of 95 °C for 1 s and 60 °C for 20 s. To generate a standard curve for this assay, a plasmid containing the region of interest was used in serial dilutions from 5 × 10^6^ copies to five copies and a limit of detection of five copies was defined. Samples were tested triplicate in a 96-well plate format with water-only negative controls. All water controls were below the limit of detection.

### Statistics

To compare the 16S rRNA gene copy number between specimens, CT values from qPCR assay were converted to copy numbers as determined by the standard curve [*y* = − 4.4358*x* + 41.126, *R*^2^ = 0.9941]. Copy number was normalized to input volume, and a non-parametric Mann-Whitney test (two-tailed) was performed to compare the 16S rRNA gene copy number between specimen types. The bacterial OTU richness was rarefied from 10,000 to 50,000 sequencing reads, in steps of 10,000 over 10 iterations each. To compare the bacterial microbiota beta diversity between specimen groups, Mann-Whitney test (two-tailed) was performed. The heatmap of virome abundance was plotted in R using gplots, with clustering by virus abundance (rows).

## Additional files

Additional file 1: **Table S1.** Summary of demographics (DOCX 13 kb)

Additional file 2: Metadata associated with all samples used in this study. (TXT 1 kb)

Additional file 3: 16S operational taxonomic unit table. (CSV 40 kb)

Additional file 4: Fasta sequence file of most abundant OTUs only detected in amniotic fluid. (FA 5 kb)

Additional file 5: Virome species table. (CSV 73 kb)

## Abbreviations

CMV: Cytomegalovirus
HPV: Human papilloma viruses
MDA: Multiple displacement amplification
SIA: Sequence-independent DNA and RNA amplification
