# Correction to: Amniotic fluid from healthy term pregnancies does not harbor a detectable microbial community

**DOI:** 10.1186/s40168-019-0641-6

**Published:** 2019-02-12

**Authors:** Efrem S. Lim, Cynthia Rodriguez, Lori R. Holtz

**Affiliations:** 1 School of Life Sciences, Arizona State University, Tempe, AZ 85287 USA; 2 The Biodesign Institute, Center for Fundamental and Applied Microbiomics, Tempe, AZ 85287 USA; 3 Department of Pediatrics, Washington University School of Medicine, St. Louis, MO 63110 USA

**Correction to: Microbiome (2018) 6:87**

**https://doi.org/10.1186/s40168-018-0475-7**

Following publication of the original article [1], the authors reported a typographic error in the type of master mix kit used, the text should read as:

“The qPCR was performed using Fast SYBR Green Master Mix (Thermo Fisher).”

The authors regret this error and the inconvenience caused.
