# Supplementary material for: Amniotic fluid from healthy term pregnancies does not harbor a detectable microbial community
Source: Microbiome. 2018 May 11;6:87. doi: 10.1186/s40168-018-0475-7 (PMC5946436; doi:10.1186/s40168-018-0475-7)
Supplement: Supplementary file 1 — Table S1. Summary of demographics (DOCX 13 kb) [file 40168_2018_475_MOESM1_ESM.docx]

| Table S1. Summary of Demographics | |
| --- | --- |
| Median Maternal Age | 28 years |
| Maternal Race (White) | 11 (46%) |
| Median Infant Birth Weight | 3287.5 grams |
| Infant Gender (Male) | 14 (58%) |
